# Modeling and Molecular Dynamics Studies of Flavone―DENV E-3 Protein―SWCNT Interaction at the Flavonoid Binding Sites

**DOI:** 10.3390/v17040525

**Published:** 2025-04-04

**Authors:** Cecilia Espíndola

**Affiliations:** Department of Physical Chemistry, University of Seville, C/Profesor García González 1, 41012 Seville, Spain; carespdia@alum.us.es

**Keywords:** flavones, DENV E-3 protein, antiviral pharmacology, non-covalent interaction, molecular dynamic simulation, docking molecular, Flavone—DENV E-3 interactions, SWCNT–flavonoids, anti-DENV drugs, nanomedicine

## Abstract

The DENV virus circulates freely in endemic regions and causes dengue disease. The vectors are *Aedes aegypti* and *Aedes albopictus*. The difficulties inherent in the nature of the DENV virus, its epidemiology, and its increasing incidence in recent years have led to the development of viable alternatives in the search for effective solutions for the treatment of this severe disease. Flavones such as tropoflavin, baicalein, and luteolin have anti-DENV activity. Molecular docking studies were performed between the flavones tropoflavin, baicalein, and luteolin and the DENV E-3 protein. Flavone—DENV E-3 complex interactions were analyzed at the flavonoid binding sites domain I of the B chain and domain II of the A chain reported in the literature. H-bond, π-π stacking, and π-cation interactions between flavones and the DENV E-3 protein at different binding energies were evaluated. Molecular dynamics studies for these interactions were performed to determine the molecular stability of the Flavone—DENV E-3 complexes. I also present here the results of the molecular interactions of the Flavone—DENV E-3―SWCNT complex. Due to recent advances in nanotechnology and their physicochemical properties, the utilization of nanoparticles such as SWCNT has increased in antiviral drug delivery.

## 1. Introduction

The dengue virus (DENV) is transmitted to humans through the bite of infected mosquitoes, usually in tropical and subtropical climates throughout the world, particularly in urban and semi-urban areas. Dengue is a viral infection caused by the DENV virus transmitted by the bite of this mosquito, which causes severe clinical symptoms such as fever and circulates through the bloodstream causing hemorrhage, hypovolemic shock and even death. The main vectors transmitting the disease are *Aedes aegypti* and, to a lesser extent, *Aedes albopictus* mosquitoes, although in some regions such as Europe and North America the latter vector is more widespread [1]. DENV-3 virus is considered to have originated around 1890 [2].

A global dengue DENV incidence analysis revealed that in more than 80 countries/territories across various regions such as Africa, the Eastern Mediterranean, European, Southeast Asia, the Western Pacific, and the Americas, more than five million cases and more than 5000 dengue-related deaths have been reported, a figure close to the historical maximum reported since the beginning of 2023. In Africa, 171,991 cases of dengue have been reported in countries in the region in 2023, as well as 753 deaths. Since 1998, both epidemic and severe dengue cases have been reported in the Eastern Mediterranean region. In 2023, of the endemic countries Somalia, Pakistan, Saudi Arabia, Afghanistan, Yemen, Egypt, Djibouti, Oman, and Sudan, the highest number of confirmed cases was reported from Pakistan, Saudi Arabia, and Oman. In this region, all four serotypes circulate. Although *Ae. aegypti* is the predominant vector transmitting dengue in countries in this region, the secondary dengue vector *Ae. albopictus* has also been reported. Dengue is not endemic to the European region, but autochthonous cases have been reported since 2010 in countries such as Croatia, France, Israel, Italy, Portugal, and Spain. Furthermore, sporadic cases and outbreaks were reported in Italy, France, and Spain in 2023. *Ae. albopictus* is established in southern Europe; however, in recent years, it has been detected further north and west. *Ae. aegypti* does not survive the winter well but has been established in Cyprus and Madeira (Portugal) since 2022. Many countries in the Southeast Asia region, including Bangladesh and Thailand, reported a marked increase in dengue cases in 2023, with India, Indonesia, Myanmar, Sri Lanka, and Thailand being among the 30 most endemic countries in the world. Bangladesh reported 308,167 cases and 1598 deaths that year, while Thailand reported 136,655 cases and 147 deaths. More than 500,000 dengue cases and 750 deaths were reported in the Western Pacific region in 2023 in the eight countries that make up the region: Australia, Cambodia, China, Lao People’s Democratic Republic, Malaysia, the Philippines, Singapore, and Vietnam. The most affected countries were the Philippines with 167,355 cases and 575 deaths and Vietnam with 149,557 cases and 36 deaths. The Americas region reported nearly 80% of the total cases reported worldwide in 2023, or 4.1 million; of these cases, 45% were laboratory-confirmed. Colombia reported the highest number of severe cases (1504), followed by Brazil (1474), Mexico (1272), Peru (1065), and Bolivia (640). The changing distribution of vectors, mainly *Aedes aegypti* and *Aedes albopictus*, especially in countries where dengue was not present, the effects of the El Niño phenomenon in 2023, and consequences of climate change such as higher temperatures, rainfall, and humidity, among others, are the factors associated with the increase in the spread of the dengue epidemic, a cyclical epidemic that recurs every three to five years [3].

DENV-1 and DENV-2 serotypes for several years were the predominant serotypes detected in the region; however, DENV-3 and DENV-4 serotypes were detected more frequently in 2023. Although all four serotypes currently circulate in the Americas, it is important to note that all four serotypes simultaneously circulate in countries such as Guatemala, Costa Rica, Mexico, Honduras, Nicaragua, Panama, Colombia, Brazil, and Venezuela [2].

**Figure 1:**
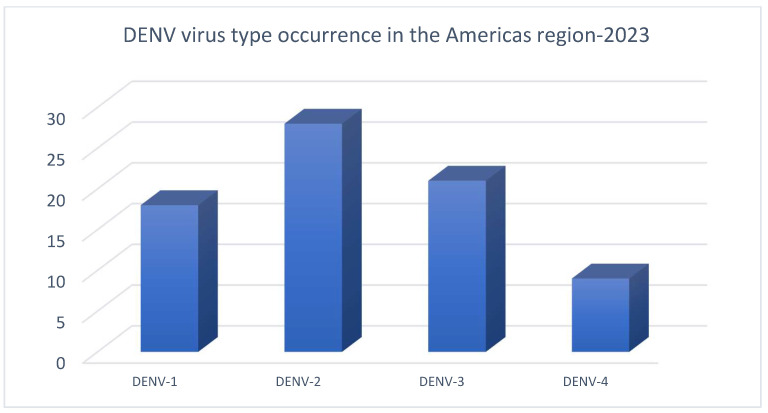
DENV virus types in Americas region in 2023 [3].

From the data obtained on the presence of the different serotypes of DENV in the Americas region, the highest occurrence was found to be serotype DENV-2, followed by serotype DENV-3, DENV-1, and DENV-4 (Figure 1).

The DENV-3 serotype was first reported in 1953 in Asia (Philippines and Thailand), in 1963 in America (Puerto Rico), and 1984–1985 in Africa (Mozambique) [4]. The different serotypes share approximately 65% similarity in amino acid sequences. In turn, each serotype includes several genotypes. The DENV-3 serotype includes four genotypes: genotype I includes strains from Indonesia, Malaysia, Philippines, and South Pacific Islands; genotype II includes strains from Thailand, Vietnam, and Bangladesh; genotype III has strains from Sri Lanka, India, Africa, Samoa, and Thailand; and genotype IV includes strains from Puerto Rico, Latin America, Central America, and Tahiti [5].

Naveca et al. (2023) [6] reported the finding of new cases of DENV-3 in northern Brazil, associated with genotype III from the Indian continent. Similarly, Adelino et al. (2024) [7] reported the resurgence of new cases of DENV-3 in Minas Gerais, Brazil. However, despite the high presence of DENV type 3, there are few reports of both in vitro and in silico studies.

**Figure 2:**
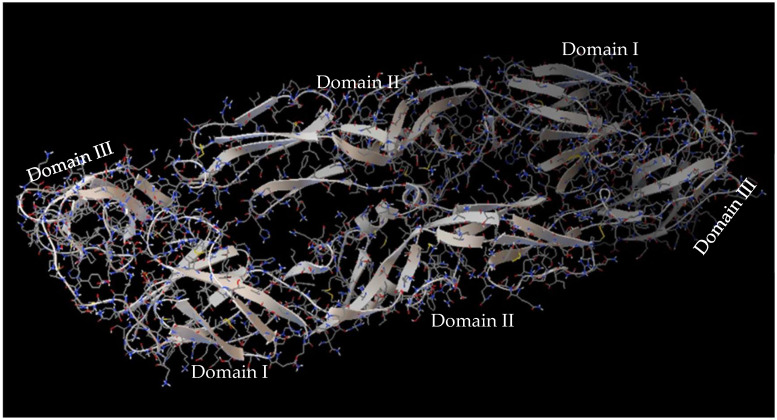
DENV E-3 protein.

The DENV virus belongs to the Flaviviridae family, consisting of four serotypes DENV-1, DENV-2, DENV-3, and DENV-4, which cause dengue disease and circulate freely in different endemic areas. The DENV virus contains a monocatenary linear RNA encoding structural proteins: Capside C, premembrane, and membrane (prM/M) and envelope E, as well as seven non-structural proteins such as NS1, NS2A, NS2B, NS3, NS4A, NS4B, and NS5. The E protein is a glycosylated structural protein that, together with the membrane precursor glycoprotein PrM, constitutes the structural surface of virions. The E protein plays a critical role in both neutralizing antibody elicitation as well as flavivirus biology since it facilitates membrane fusion between virus and host cell, virus entry, hemagglutination, host range, tropism, virulence, attenuation, and maturation, among others [8]. The E protein is constituted by domains I and II, which facilitate the insertion of the virus into the host cell membrane and consequent conformational change of the E protein due to pH changes, and by domain III which is responsible for the binding of the receptor [9] (Figure 2). It has been demonstrated that the E protein can agglutinate red blood cells [10]. Some molecules such as ICAM non-integrins, 2CD209, Rab 5, GRP 78, and mannose receptors present on the cell surface can interact with the DENV E protein [9].

The DENV E protein can adopt conformations in the form of a dimer or trimer. The dimer is found in the mature virion while the trimer is found in the immature state of the virion in the endosome of the host cell. There are two conserved histidine residues in glycoprotein E that can act as pH sensors, residue HIS209 and HIS7 [9,10]. The trimer transformation process is initiated when, in the endosome, histidine residues sense low pH, causing deprotonation of histidine and leading to molecular rearrangement involving domains I and II. These domains contain a hydrophobic stretch, the fusion peptide, which during the viral prefusion state is exposed and reoriented after transition to the trimeric structure of the postfusion state. Thus, in this conformation, the fusion peptide binds to the cell membrane, initiating the fusion process with the viral membrane. One important step in the production of the infectious virion occurs in the trans-Golgi network where the prM protein is cleaved by the host furin convertase protein, resulting in a piece of membrane-anchored M and a **pr** peptide that remains attached to the virus particle until secretion. Immature virions contain two associated membranes, the prM protein and the E protein, which form a tight heterodimer complex. During the virus maturation process, the prM protein cleaves, originating the rearrangement of the E protein into homodimers (mature virions). The carboxy-terminal cleavage product of prM remains associated with the viral membrane [11].

Dengue cases are usually asymptomatic or cause a low-grade febrile illness; however, the disease can become severe, causing signs such as severe abdominal pain, persistent vomiting, bleeding gums, fluid accumulation, lethargy or restlessness, and liver enlargement, followed by shock, hemorrhage, or organ damage, and finally death [12]. For dengue, there is no specific treatment since infection by one serotype provides long-term immunity against the same serotype and transient immunity against the other serotypes. Therefore, secondary infections by a different serotype increase the risk of severe dengue as recently evidenced by Tomas (2023) [13] by evaluating some vaccines developed against dengue virus. Dieng et al. (2023) [14] through phylogenetic studies evaluated the molecular evolution of DENV-3 strains circulating in Senegal during the years 2009–2022. When the amino acid sequences of the PrM and E proteins were compared with the vaccine strains, twenty-two and twenty-three amino acid sequence substitutions were found. The main substitutions were found in domain III of the E protein, a region that allows for long-lasting protective immunity against the dengue virus. Therefore, the lack of correspondence between circulating strains and vaccines is one of the main causes of the low efficiency of existing vaccines.

Alternatives are being developed for the treatment and prevention of the disease caused by the DENV virus [15], including the utilization of the pharmacological potential of some flavonoids which can alter the replication of various types of viruses such as DENV [16]. In nature, flavonoids are found as chemical compounds with biological and physiological functions in both plants and animals [17]. They are products of secondary metabolism in plants that are important for developmental processes and as defense mechanisms against bacteria, viruses, and diseases, among others. The chemical structure of a flavonoid is a skeleton of 15 carbon units forming two benzene rings A and B joined by a heterocyclic pyrene ring C. The pharmacological activity of a flavonoid is directly related to its chemical nature, which depends on its structure, the degree of hydroxylation, substitution, conjugation, and the degree of polymerization, among others [18]. Among the flavonoids we can find flavones, a group of flavonoids with the B ring connected in the C2 position, a double bond between C2 and C3, an oxidized C4 atom, and no substitution in the C3 position; this chemical structure gives them their biological and pharmacological properties [19]. Flavones can be presented as aglycones or glycones.

Among the flavones with antiviral activity are *7*,*8-dihydroxyflavone*-tropoflavin, *5*,*6*,*7*,*-trihydroxyflavone*-baicalein and *3′*,*4′*,*5*,*7*,*-tetrahydroxyflavone*-luteolin (Supplementary Material Figure S1) whose anti-DENV activity has been demonstrated by in vitro assays. Tropoflavin is a flavone with hydroxyl groups at the -7 and -8-OH positions. Tropoflavin has been shown to have anti-enterovirus EV71 activity, interfering with virus replication [20,21]. Baicalein is a trihydroxyflavone with hydroxyl groups at the -5, -6, and -7-OH positions. Baicalein has anti-DENV activity. The ability of baicalein to bind and inactivate structural and non-structural proteins of the DENV virus could explain the effectiveness of baicalein as an anti-DENV compound. Thus, Zandi et al. (2012) [22], evaluating the anti-dengue activity of baicalein, found that after adsorption, baicalein inhibited DENV-2 replication in Vero cells (IC_50_: 6.46 μg/mL) and presented direct anti-DENV-2 virucidal activity with an IC_50_ of 1.55 μg/mL. In turn, Low et al. (2021) [23] evaluated the anti-DENV effect of baicalein on the four serotypes DENV-1, DENV-2, DENV-3, and DENV-4. Baicalein inhibited DENV-3 by direct extracellular virucidal action, 62.45% at 0 min and advanced to 97.24% at 60 min. At a dose of 100 μg/mL, it blocked virus binding to Vero cells in 95.59%, inhibited virus entry into cells in 57.91%, and neutralized the DENV-3-infected cell population in 97.92%. Similarly, it inhibited viral infectivity of DENV-1 in 96.68% of DENV-2 in 97.16%, of DENV-3 in 99.68%, and of DENV-4 in 80.49%. Luteolin is a tetrahydroxyflavone with hydroxyl groups at the 3′,4′,5,7-OH positions. Peng et al. (2017) [24] reported the anti-dengue activity of luteolin against all four DENV serotypes: type 1 (EC_50_: 4.36 μM; SI: 10.53, Vero cells), type 2 (IC_50_: 5.19 mM SI: 8.84), type 3 (EC_50_: 5.69 μM; SI: 8.07, Vero cells), and type 4 (EC_50_: 8.38 μM; SI: 5.48, Vero cells) after 48 h of incubation. Luteolin inhibits viral maturation processes after entering the cell [25].

Among the main objectives of molecular docking is the identification of a ligand, its binding site to the protein, and its energetically most favorable binding mode. In silico, there are few studies on the evaluation of anti-DENV activity. Ismail and Jusoh (2017) [26] evaluated the interaction of flavonoids with the DENV protein. Flavonoids baicalein, baicalin, fisetin, flavone, glabranin, hyperoside, ladanein, and quercetin were utilized to determine their binding site to the DENV E protein by molecular docking. In silico studies previously performed by Espíndola (2023) [27] analyzed the types of molecular interactions between flavones tropoflavin, baicalein, and luteolin and the DENV E-3 protein. In these studies, the importance of the -OH group in the formation of H-bond, π-cation, and π-π stacking bonds with different amino acids of the DENV E-3 protein and their corresponding angles and distances were highlighted.

Due to recent advances in nanotechnology and their physicochemical properties, nanoparticles, such as CNTs [28], have been utilized in antiviral drug delivery [29]. Carbon nanotubes (CNTs) are graphene sheets rolled into long hollow cylindrical structures with diameters between 1 and 10 nm. There are mainly single-walled (SWCNTs) and multi-walled (MWCNTs) carbon nanotubes [30]. Properties such as their large modifiable surface area, loading efficiency, physicochemical properties, ability to be functionalized, biocompatibility with nature, cell internalization via endocytosis, and low cytotoxicity, among others, enable their application in the delivery of drugs and small molecules with anticancer, anti-inflammatory, antioxidant, antimicrobial, and antiviral activity, among others [31,32]. 

Research on flavone encapsulation in pristine and functionalized SWCNTs and MWCNTs has been developed. Flavone encapsulation parameters such as size, PDI, ζ-potential, EE, and flavone release in vitro have been determined. The chemical structure of flavones seems to play a key role in the interactions, although other types of interactions are also involved [33]. Encapsulation studies of 7,8-dihydroxyflavone in SWCNTs and MWCNTs have been developed, where the effect of factors such as pH and type and concentration of the CNT on the association processes have been considered. Furthermore, some of the activities of flavones such as the antioxidant capacity of free and encapsulated flavones in CNT/flavone complexes were also determined [34].

CNTs can also be utilized as nanocarriers of both antiviral agents (flavones) and virus for the development of anti-DENV E-3 vaccines [35,36]. Research carried out with CNTs functionalized with nanoconjugates of the recombinant envelope of dengue virus DENV E-3 found that they could induce immune responses specific to serotype 3, but with prospects for proteins derived from the other dengue serotypes [37]. Similarly, when CNTs were utilized to increase the expression of a tetravalent dengue vaccine candidate plasmid in in vitro and in vivo studies, it was demonstrated that plasmid-associated CNTs are internationalized by cells and subsequently dispersed in the cytoplasm and nucleus [35,38].

I present in this work some results of molecular docking studies involving flavones tropoflavin, baicalein, and luteolin as antiviral agents and the DENV E-3 protein by analyzing the main molecular interactions at the flavonoid binding sites. The results of some studies on the interactions of Flavone—DENV E-3-SWCNT complexes, in which CNTs are utilized as nanotransporters of these antiviral agents, are also presented. Molecular dynamics studies of Flavone—DENV E-3 interactions have been performed and the analysis of these is presented here.

## 2. Materials and Methods

The ligands utilized were *7*,*8-dihydroxyflavone* (tropoflavin; PubChem ID 1880); *5*,*6*,*7- trihydroxyflavone* (baicalein PubChem ID 5281605); and *3′*,*4′*,*5*,*7- tetrahydroxyflavone* (luteolin PubChem ID 5280445). The DENV E type 3 protein was obtained from Protein Data Bank (PDB) crystal structure code 1UZG (https://www.rcsb.org/3d-view/1UZG/1, accessed on 2 April 2025). In this work, Autodock 4.0 and Autodock vina 4.0 in molecular docking and Desmond Schrödinger’s Desmond (version 2021.4) in MD have been utilized for the study and analysis of Flavone—DENV E-3 interactions taking into account the accuracy of binding modes and affinity energies obtained with computational docking.

### 2.1. Docking Protocol

In Autodock, the semi-empirical force field includes intramolecular terms and a full solvation model and considers the directionality of hydrogen bonds. The best binding mode considers the binding energy (lowest), the inhibition constant (lowest), and the highest number of H-bond interactions with the active site of the protein. An approximation is made for the free energy function utilizing the thermodynamic cycle of Wesson and Eisenberg:

$$∆G=∆G_{vdW}\sum_{i,j}\left(\frac{A_{ij}}{r_{ij}^{12}}-\frac{B_{ij}}{r_{ij}^{6}}\right)+∆G_{hbond}\sum_{i,j}E\left(t\right)\left(\frac{C_{ij}}{r_{ij}^{12}}-\frac{D_{ij}}{r_{ij}^{10}}\right)\;+∆G_{elec}\sum_{i,j}\frac{q_{i}q_{j}}{\varepsilon \left(r_{ij}\right)r_{ij}}\;+∆G_{tor}N_{tor}\;+∆G_{sol}\sum_{i_{,}j}(S_{i}V_{j}+S_{j}V_{i\;}{)e}^{\left(-r_{ij}^{2}/2\sigma^{2}\right)}$$

where Δ*G* is the free energy to be minimized; Δ*G_vdW_* is the dispersion and friction energy; Δ*G_hbond_* is the H-bond energy; Δ*G_elec_* is the electrostatic energy; Δ*G_tor_* models the constraint of the internal rotations; and Δ*G_sol_* explains the hydrophobic effect of the coupled structure [39].

In turn, AutoDock Vina improves the precision of conformational predictions, calculates energy maps, and clusters the results. Vina utilizes a variant of X-score [40] and a scoring function that considers inter- and intramolecular contributions.

The scoring function for Vina is defined as follows:

$$C=\sum_{i<j}f_{t_{i}t_{j}}\left(r_{ij}\right)$$

where the sum includes pairs of atoms that can move to each other and excludes atoms separated by up to three consecutive covalent bonds. *t_i_* means the type of index atom *i*, *r_ij_* is the interatomic distance, and the interaction function is defined as *ƒt_i_t_j_*(*r_ij_*) ≡ *ht_i_t_j_* (*d_ij_*), where *d_ij_* = *r_ij_* − *Rt_i_* − *Rt_j_*. *Rt_j_* is the van der Waals radius for the atom of type *t* and is expressed in Å.

**Figure 3:**
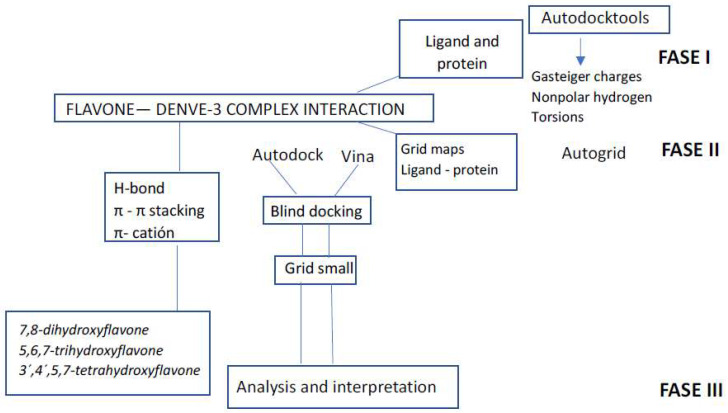
Molecular docking phases for Flavone—DENVE-3 complex interaction.

Autodock-ADT tools were utilized to prepare the DENV E-3 protein and ligands. Molecular docking was carried out with Autodock 4.0 [41] and Autodock vina 4.0 [40]. Autogrid 4.0 software was utilized to generate the energy maps. Initially, to find ligand binding sites with the DENV E-3 protein, blind docking was performed on the whole protein structure. A DENV E-3 protein grid box was carefully constructed to ensure that the entire protein was included inside the box. A second round of molecular docking was then performed according to the analysis of the binding energies of the protein–ligand complex. The grid small with the lowest energy result for each flavone was realized with a grid size of X = 58, Y = 52, and Z = 60, and a grid center of x = −5.394, y = 3.561, and z = 12.501 [29]. The phases that include the procedures carried out to perform the molecular docking of Flavone—DENV E-3 interactions are schematized in Figure 3 [27].

### 2.2. Nanotube Modeling

The 3D structure of the SWCNT for molecular docking studies of DENV E-3—SWCNT interaction and the Flavone—DENV E-3—SWCNT complex was modeled using the Nanotube Modeller program. The dimensions utilized for the SWCNT agree with those employed for medical purposes: chirality (6.5), length of 20.0 Å, diameter of 7474 Å, bond length of 1421 Å, and 261 bonds [29]. With the CRYSTAL code, the construction of the SWCNT was realized by wrapping the 2D planar structure.

In molecular docking studies of DENV E-3—SWCNT and Flavone—DENV E-3—SWCNT complexes, Autodock-ADT tools were utilized for ligand, protein, and SWCNT preparation. For SWCNT ligand preparation, the center of mass was determined, Gaisteger Marsili charges, the number of rotating atoms, and the number of spins were assigned, and the output file was identified as pdbqt [29]. Blind docking with vina for the DENV E-3—SWCNT interaction was realized with a grid box with the following dimensions: X = 88, Y = 74, and Z = 200. Grid center dimensions were x = 11.939, y = −2.391, and z = 1.398. Molecular docking for DENV E-3—SWCNT and Flavone—DENV E-3—SWCNT complex interactions was performed with Autodock and Autodock Vina following the methodology described for the interaction between Flavone—DENV E-3 complexes (Figure 3) [27].

### 2.3. Molecular Dynamics

Molecular dynamics uses algorithms from computer science and information theory. It allows us to understand materials and molecules not as rigid entities but as animated bodies. It has also been called “numerical mechanical statistics” or the Laplace view of “Newtonian mechanics” since it aims to animate the forces of nature [42].

The aim of biomolecular simulations is to achieve the accurate and predictive computer simulation of the physical properties of biological molecules in their aqueous environments. Models are determined from quantum mechanics, molecular mechanics, experimental results, and these combinations [43]. Among the water models are three-point charge models. In molecular simulation, formal statistical mechanics designations for ensembles, are utilized. They imply the variables of the thermodynamic system that are regulated or conserved [44]. In all ensembles, the number of particles (N) is conserved. In the case of NPT, the simulation of the system is based on NVT, but the pressure (P) is regulated [45]. The Nosé–Hoover thermostat is a deterministic algorithm for constant temperature molecular dynamics simulations. Therefore, the Nosé–Hoover thermostat has been commonly used as one of the most accurate and efficient methods for constant temperature molecular dynamics simulations [46,47].

RMSD (root mean square deviation) is the measure of the average distance between atoms of overlapping ligand–protein complexes used to study the configuration of ligands when they bind to proteins. It is calculated for all frames of the trajectory.

The RMSD for conformation *x* is as follows:

$${RMSD}_{X}=\sqrt{\frac{1}{N}\sum_{i=1}^{N}\left(r_{i}^{\prime }\left(t_{x}\right)\right)-r_{i\;}{\left(\left(t_{ref}\right)\right)}^{2}}$$

where *N* is the number of selected atoms; *t_ref_* is the reference time; and *r’* is the position of the selected atoms in conformation *x* after superimposing it on the reference structure, where conformation *x* is recorded at time *t_x_*. The procedure is repeated for each frame of the simulation trajectory [48].

Molecular dynamics studies of a protein–flavonoid complex are generally applied to the search for the conformational stability of the protein upon ligand binding [49]. RMSD is a measure of the structural stability of the protein as the simulation time progresses.

The root mean square fluctuation (RMSF) of the protein is useful for characterizing local changes along the protein chain.

The RMSF for residue *i* is as follows:

$${RMSF}_{X}=\sqrt{\frac{1}{T}\sum_{t=1}^{T}<\left(r_{i}^{\prime }\left(t\right)\right)-r_{i\;}{\left(\left(t_{ref}\right)\right)}^{2}>}$$

where *T* is the trajectory time on which the RMSF is calculated, *t_ref_* is the time of reference, *r_i_* is the position of residue *I*, *r’* is the position of atoms in residue *i* after superposition on the reference, and the angle brackets mean that the squared distance is based on a selection of atoms from the residue. In molecular dynamics, the protein–ligand interactions encountered are hydrogen bonds, hydrophobic bonds, ionic bonds, and water bridges [48].

The results of molecular docking between flavones tropoflavin, baicalein, and luteolin and the DENV E-3 protein whose interactions correspond to flavonoid binding sites with the DENV E protein reported by Ismail and Jusoh, 2017 [26] were selected for molecular dynamics studies. These interactions were GlyA:152—tropoflavin, with Δ*G* = −7.0 kcal/mol, ArgA:99—baicalein, with Δ*G* = −3.3 kcal/mol, and LysA:245—luteolin, with Δ*G* = −5.19 kcal/mol (Supplementary Material Table S1).

Simulation was performed with Schrödinger’s Desmond program (version 2021.4) [48] to verify the stability and dynamics of Flavone—DENV E-3 interactions. The stability of Flavone—DENV E-3 interactions was confirmed by analyzing the trajectories in the root mean square deviation (RMSD) of the protein and ligand, the root mean square fluctuation (RMSF) of the protein and ligand, H-bonding, hydrophobic π-cation interactions, π-π stacking, and water bridges.

To conduct molecular dynamics studies of Flavone—DENV E-3 complexes, the flavone–protein complexes were first prepared, the simulation environment was constructed, and the solvation parameters were defined: box triclinic, a buffer distance of 10 Å, the SPC model, a van der Waals cutoff of 1.0 nm, system neutralization with 2Na^+^, [NaCl] 0.1 M, and a force field of OPLS 4. The system was balanced with ensemble NPT, and the Nose–Hoover thermostat and the Martyna–Tobias–Klein barostat were applied for constant temperature (300 K) and pressure (1 bar). The simulation was run for 100, 200, and 500 ns for Flavone—DENV E-3 complexes. The simulation was run with a 2 fs time step. The simulation trajectory was carried out with Desmond software [26,29].

## 3. Results and Discussion

### 3.1. Interaction of DENV E-3–Flavone at Binding Site of Flavonoids

Previous studies on the interactions of flavonoids with the DENV E-2 protein indicate that the binding site of some flavonoids is in the residues of domain I of the B chain (residues 4–9, 151–154) and domain II of chain A (residues 98–103, 244–247), which includes the conserved region of the fusion peptide. Similarly, it has been found that the region where the fusion peptide is formed (residues 98–108) is a highly conserved region among flaviviruses. Although the DENV E-3 protein has a high similarity with the DENV E-2 protein, the flavonoids that are coupled with the DENV E-3 protein have very different results from those of the other E proteins evaluated. Baicalein showed coupling in another chain different from the other flavonoids evaluated for interaction with DENV E-3. The explanation for this different response lies in the different orientation of domains I and II of the crystallized DENV E-3 protein (1UZG.pdb) [26].

In the molecular docking of the baicalein–DENV E-3 complex with vina, it was found that with Δ*G* = −3.3 kcal/mol there is an H-bond interaction with ArgA:99 and 2.45 Å in domain II of the A chain, the region in which the fusion peptide is included. In this interaction, it is the NH_2_ group of ArgA:99 that donates H to the *4-oxo* group of baicalein (Table 1). This interaction has close contact with the following residues: ValA:97, AlaA:243, LysA:244, HisA:242, GlyB:47, GlyB:5, ValB:6, AspA:98, and AsnA:103.

Table 1 also shows that tropoflavin has an H-bond interaction with LysA:245 at Δ*G* = −4.49 kcal/mol. This interaction occurs between the alkylammonium ion (―CH_2_―NH_3_^+^) of LysA:245 and the group 7-OH of tropoflavin at 2.42 Å, where it is the alkylammonium ion that donates the H to the 7-OH group of the flavone. At this same value of Δ*G*, the interaction of the H-bond with AlaA:241 is also present, where it is the NH_2_ group of AlaA:241 that donates H to the *4-oxo* group of the *7,8-dihydroxyflavone* C ring with 2.0 Å. These interactions have close contact with the residues LysA:244, GluA:247, AsnA:240, LysA:239, IleB:276, and HisA:242.

**Figure 4:**
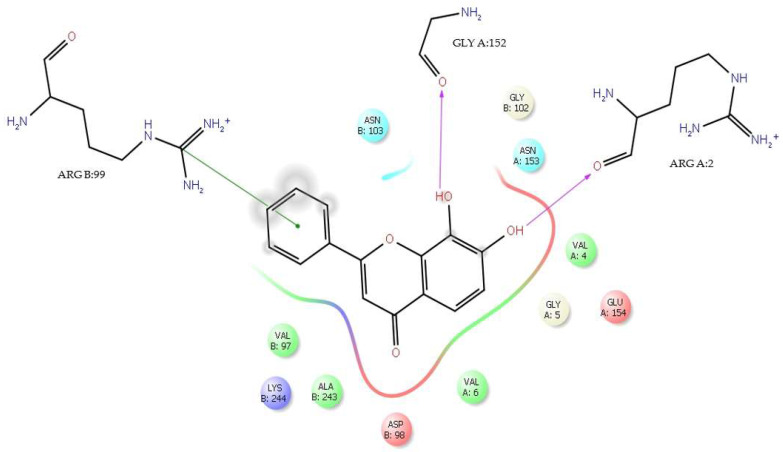
H-bond interaction of residues GlyA:152, ArgA:2 and π-π stacking interaction of residue ArgB:99 with tropoflavin. Δ*G* = −7.0 kcal/mol.

It is important to note that tropoflavin in the interaction with the residue Gly:152 at Δ*G* = −7.0 kcal/mol (Table 1) presents Asn153 as a close contact. The interaction of tropoflavin with Gly:152 is carried out by means of an H-bond in which it is the 8-OH group of the flavone A ring that donates the H to the keto group of GlyB:152 with 2.37 Å. Similarly, there is a π-π stacking interaction between the tropoflavin C ring and the protonated guanidinium ion (- C- (NH_2_)_2_^+^) of ArgB:99 at 5.36 Å. There is also an H-bond interaction between the 7-OH group of the A ring and ArgA:2 where it is the 7-OH group that donates the H to the keto group of ArgA:2 at 2.27 Å (Figure 4). In these interactions, tropoflavin also has close contact with GlyB:102, AsnB103, ValB:97, LysB:244, AlaB:243, AspB:98, ValA:5, GlyA:5, ValA:4, and GluA:154.

The dengue virus has two conserved *N*-glycosylation sites. The *N*-glycosylation site in Asn153 is conserved in most flaviviruses, while Asn67 appears in only a few. Both glycans are involved in host cell binding and virus entry [9,26,50,51,52,53,54,55]. The side chains of Asn are electrically neutral; however, it is a very polar amino acid and is frequently found on the surface of proteins. The polar amide groups of Asn also form hydrogen bonds with side chains of other amino acids [56]. Mondote et al. (2007) [57] studied the role of *N*-glycosylation at different stages of DENV viral replication and found that glycan at the Asn153 position slightly increases the production of viral particles in the virus but dramatically increases viral infectivity in mammalian host cells.

The dengue virus infects dendritic cells, monocytes, and macrophages, essential for inducing and maintaining the innate and adaptive immune response [58]. It has been proposed that immature dendritic cells are the first targets after the virus is introduced into the bloodstream. Similarly, it has been found that the glycans present in the E protein of the virus are involved in binding to the DC-SIGN receptor, a C-type lectin receptor that is essential for producing infection and is present on the surface of macrophages and dendritic cells [52,54,55].

**Figure 5:**
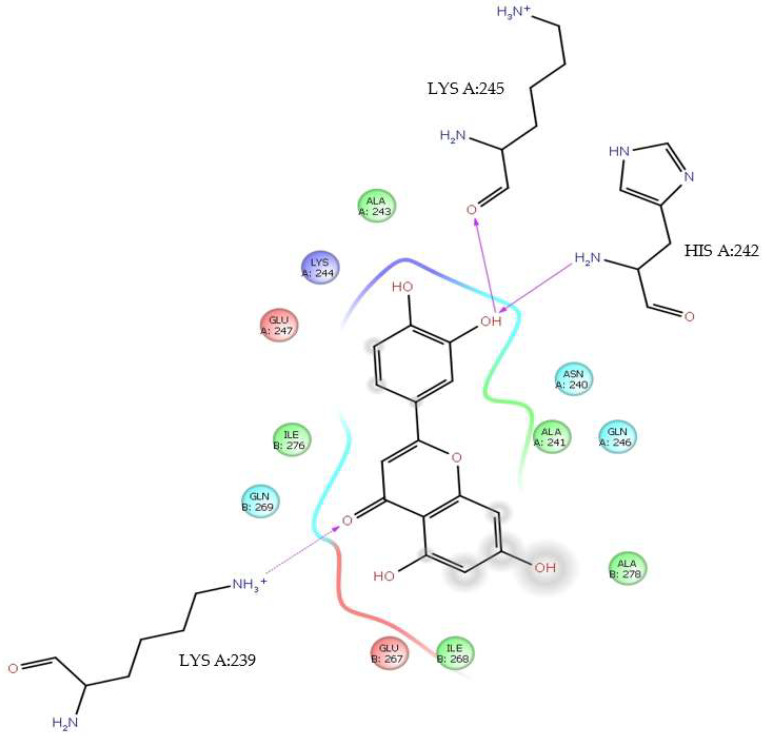
H-bond interactions between luteolin and residues LysA:245, HisA:242, and LysA:239. Δ*G* = −5.19 kcal/mol.

On the other hand, luteolin also interacts with LysA:245 at Δ*G* = −5.19 kcal/mol and at 2.45 Å (Table 1). In this interaction, it is the 3′-OH group that donates the H to the keto group of LysA:245. With the same value of Δ*G*, this same group 3′-OH receives an H from the NH_2_ group of HisA:242 with 2.66 Å in an H-bond interaction. In addition, the *4-oxo* group of the luteolin C ring in an H-bond interaction receives the H of the alkylammonium ion (―CH_2_―NH_3_^+^) from LysA:239 at 2.03 Å. These interactions have close contact with LysA:244, AlaA:243, AsnA:240, AlaA:241, GlnA:246, AlaB:278, GluB:267, IleB:268, GlnB:269, IleB:276, and GluA:247 (Figure 5).

In addition to the H-bond interactions between the tropoflavin, baicalein, and luteolin complexes with the DENV E-3 protein evaluated by us, which coincide with the binding sites for the flavonoids reported by Ismail and Josuh (2017) [26], there are also π-cation interactions with baicalein (Table 2).

**Figure 6:**
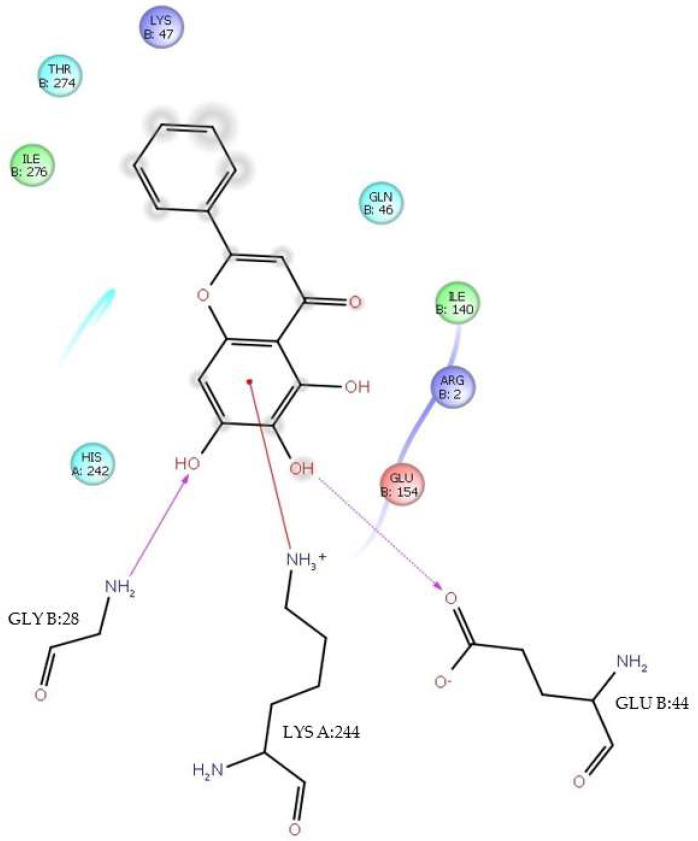
H-bond and π-cation interactions between baicalein and residues GluB:44, GlyB:28 and LysA:244. Δ*G* = −6.4 kcal/mol.

A π-cation interaction occurred between baicalein and LysA:244 at Δ*G* = −6.4 kcal/mol and 3.72 Å. This interaction occurs between the A ring of the flavone and the alkylammonium ion (—CH_2_—NH_3_^+^) group of LysA:244. The π-cation interaction between baicalein and LysA:244 has close contacts to the residues IleB:276, ThrB:274, LysB:47, GlnB:46, IleB:140, ArgB:2, GluB:154, GluB:44, GlyB:28, and HisA:242. It should be mentioned that at this ΔG value, in addition to the π-cation interaction, there is also an H-bond interaction between the 6-OH group of the A ring and GluB:44, where it is 6-OH who donates the H to the keto group of the carboxyl group γ at 1.82 Å. In addition, there is also an H-bond between the 7-OH group of the same ring and GlyB:28, where it is the NH_2_ group of the residue that donates the H to the 7-OH group of the flavone at 2.37Å (Figure 6).

It is important to highlight in previous studies [27], at Δ*G* = −7.05–7.18 kcal/mol, baicalein presented π-π stacking interactions with Trp:229 and H-bond interactions with Gln:120, where π-π stacking distances were higher (5.27–5.47 Å) than H-bond distances (2.50–2.56 Å).

### 3.2. Flavone—DENV E-3—SWCNT Complex Interaction

**Figure 7:**
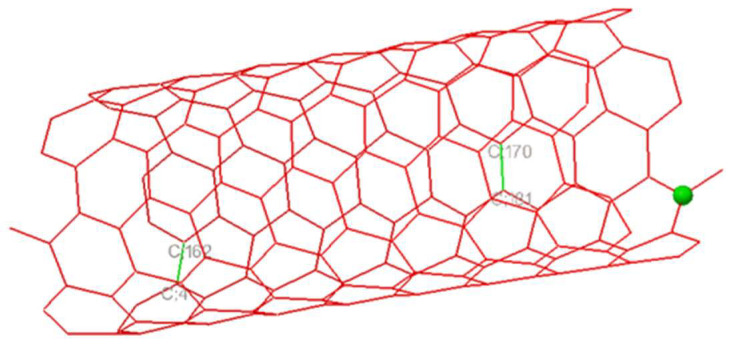
The rotatable bonds in the SWCNT.

**Figure 8:**
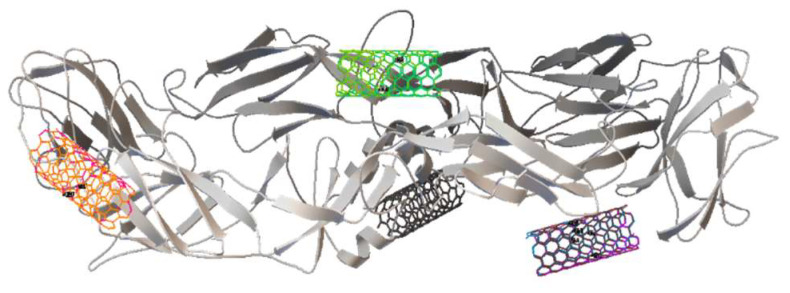
SWCNT positions on the DENV E-3 surface.

In the SWCNT ligand preparation for both the SWCNT—DENV E-3 coupling and the Flavone—DENV E-3—SWCNT complex, the center of mass coordinates was determined to be in the SWCNT ligand, 206 nonpolar hydrogens were found, 2 rotatable bonds, and the conformational search was fit to 2. The rotatable bonds were found in C;170-C;181 and C;162-C;4 (Figure 7). Different positions of the SWCNT on the surface of the DENV E-3 protein were observed (Figure 8).

**Figure 9:**
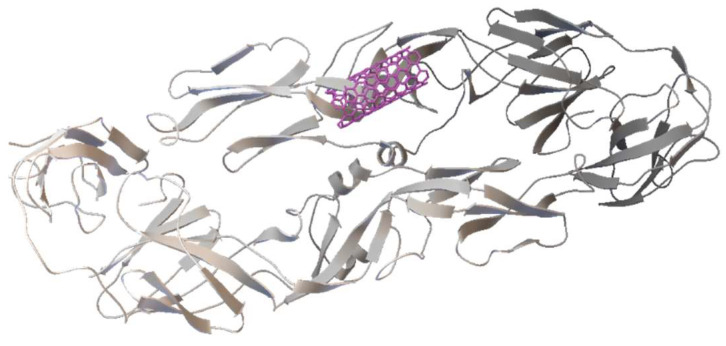
SWCNT—DENV E-3 interaction. ΔG = −11.77 kcal/mol.

In the energy histogram (Supplementary Material Figure S2), the last cluster corresponds to the energy value −11.77 kcal/mol, and at this energy value, the interaction between the SWCNT and domain II of the DENV E-3 protein occurs (Figure 9).

#### 3.2.1. Tropoflavin–DENV E-3—SWCNT

**Figure 10:**
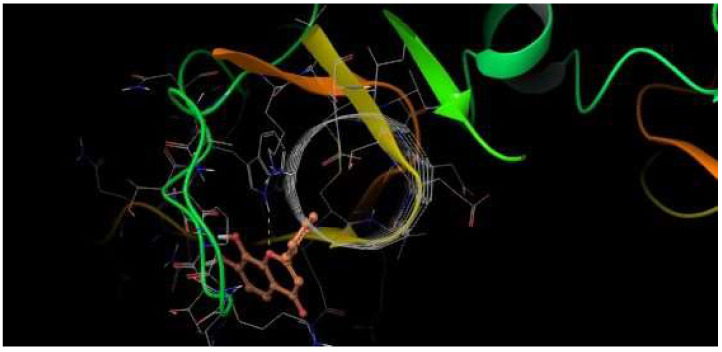
H-bond interaction with TrpB:229 in the tropoflavin–DENV E-3—SWCNT complex. Δ*G* = −6.8 kcal/mol. 3D.

The 3D model reveals the interaction that is present in the tropoflavin–DENV E-3—SWCNT complex (Figure 10). The tropoflavin–DENV E-3—SWCNT complex interaction occurs in domain II of the DENV E-3 protein. In addition to the molecular interactions described above, the complex has close contact with residues LysB:225, SerB:124, LysB:58, CysB:121, ThrB:228, PheB:119, AsnB:89, LysB:118, GlnB:120, and ThrB:226.

**Figure 11:**
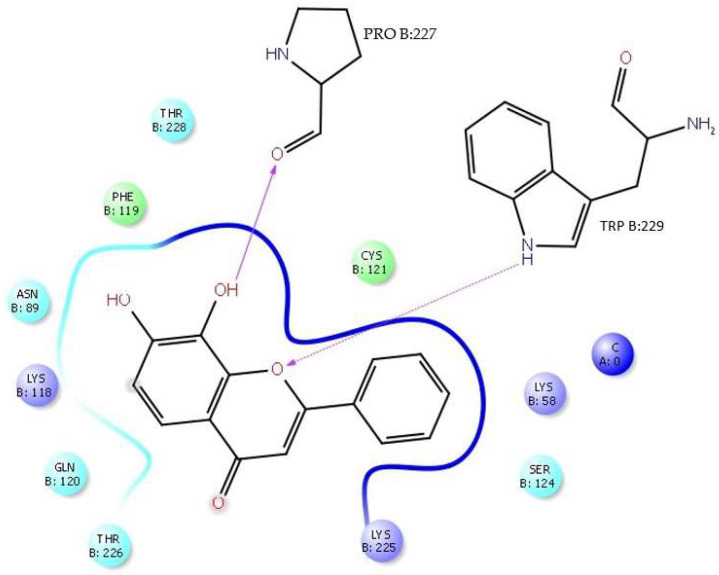
Two-dimensional diagram of H-bond interaction in tropoflavin–DENV E-3—SWCNT complex. Carbon atom of SWCNT is observed (blue).

In the interaction of the tropoflavin–DENV E-3—SWCNT complex, it is observed that tropoflavin presents an H-bond interaction with TrpB:229, where the NH of the pyrrole ring of TrpB:229 donates the H to *O*-pyran of the C ring of 7,8-dihydroxyflavone at 2.61 Å. An H-bond interaction also occurs between tropoflavin and the ProB:227 residue at 2.06 Å (Figure 11).

#### 3.2.2. Baicalein–DENV E-3—SWCNT

**Figure 12:**
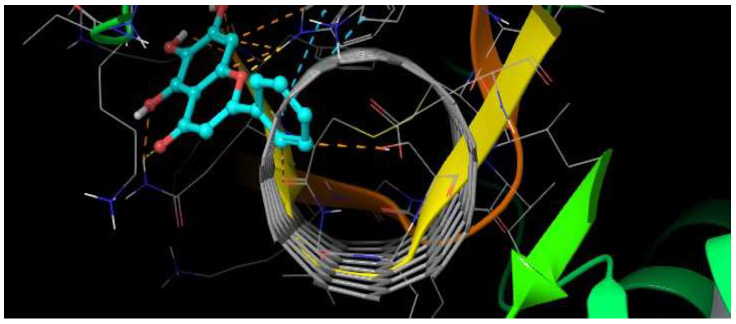
Three-dimensional diagram of π-π stacking and H-bond interactions with TrpB:229 and GlnB:120 in baicalein–DENV E-3—SWCNT complex. Δ*G* = −6.99 kcal/mol.

The baicalein–DENV E-3—SWCNT complex interaction was present at Δ*G* = −6.99 kcal/mol. In this interaction, it was observed that baicalein presents an interaction with Trp:229, with the type of π-π stacking being edge to face; this interaction occurs between the baicalein B ring and the pyrrole ring of indole group of Trp:229 with 5.47 Å. The 3D model of Figure 12 illustrates this interaction.

**Figure 13:**
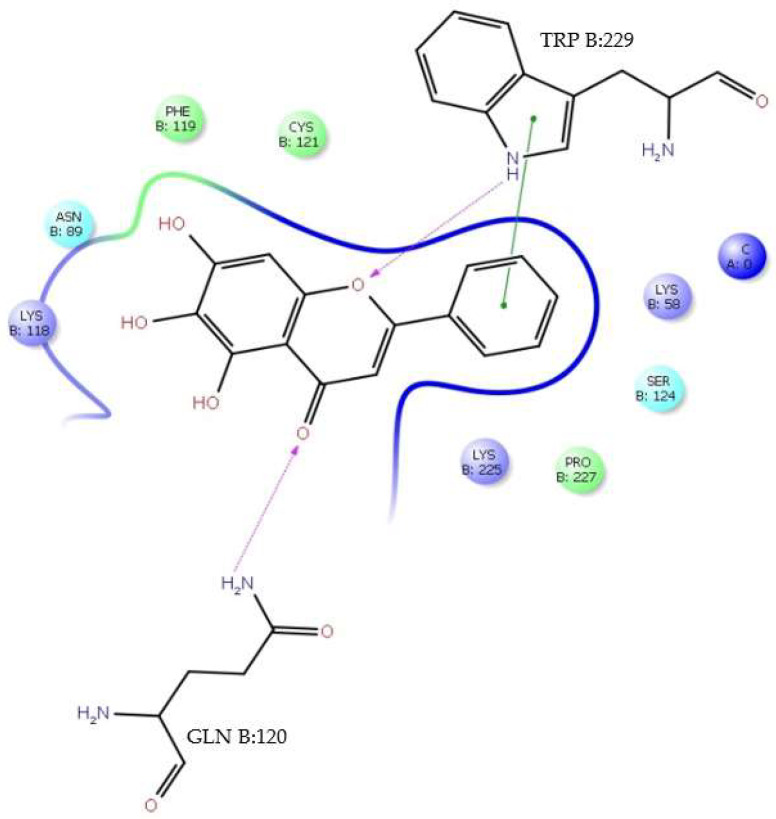
Two-dimensional diagram of π-π stacking and H-bond interaction in baicalein–DENVE-3—SWCNT complex. SWCNT is observed (blue).

In turn, the NH group of the pyrrole ring of TrpB:229 indole group presents an H-bond interaction (side chain) with the *O*-pyran of the baicalein C ring, where it is the NH group of the pyrrole ring that donates the H to *O*-pyran with 1.84 Å. The GlnB:120 residue presents an H-bond interaction (side chain) with the *4-oxo* group of the baicalein C ring, where it is the NH_2_ group of the residue that donates the H to the *4-oxo* group with 2.66 Å (Figure 13).

The baicalein–DENV E-3—SWCNT complex interaction occurs in domain II of the DENV E-3 protein. Molecular interaction of this complex has close contact with residues PheB:119, CysB:121, LysB:58, SerB:124, ProB:227, LysB:225, LysB:118, and AsnB:89 (Figure 12). In addition, it was observed that the SWCNT presents interactions with GlnB:120, CysB:121, LeuB:122, GluB:123, SerB:124, IleB:125, GluB:126, GlyB:63, LysB:64, and IleB:65 residues of the DENV E-3 protein (Figure 13).

#### 3.2.3. Luteolin–DENV E-3―SWCNT

For its part, a luteolin–DENV E-3—SWCNT complex interaction occurred between the flavone luteolin corresponding to the energy value Δ*G* = −6.8 kcal/mol and the SWCNT with Δ*G* = −11.77 kcal/mol corresponding to the energy value of the DENV E-3—SWCNT interaction. The luteolin–SWCNT complex interaction involves the *4-oxo* group of the C ring and the 5-OH group of the luteolin A ring with the SWCNT. Electrostatic interactions between the functional oxygen of the molecule and the SWCNT, van der Waals forces, and π-π stacking between the aromatic rings of the flavone and the SWCNT are involved in the flavone–SWCNT interaction [19].

**Figure 14:**
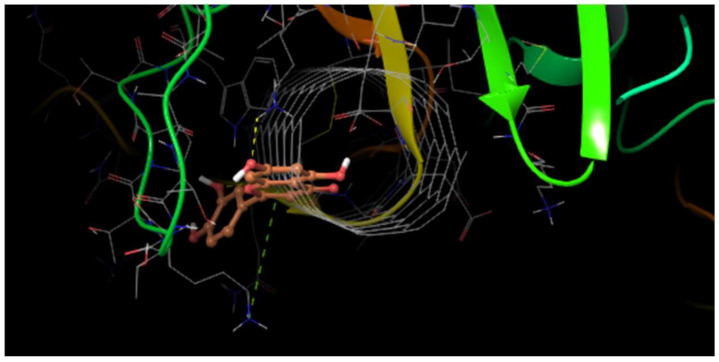
Three-dimensional diagram of H-bond interactions with LysB:58 and CysB:121 in luteolin–DENV E-3—SWCNT complex. Δ*G* = −6.8 kcal/mol.

**Figure 15:**
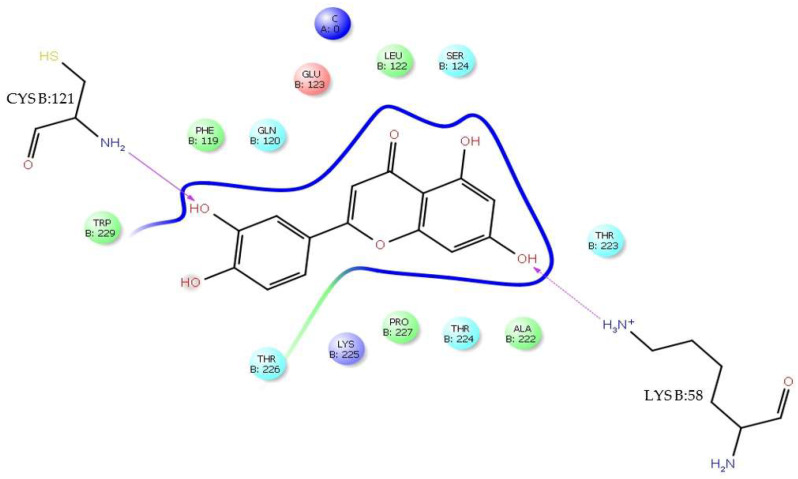
Two-dimensional diagram of H-bond interaction in luteolin–DENV E-3—SWCNT complex. SWCNT is observed (blue).

In the luteolin–DENV E-3—SWCNT complex interaction, it was observed that luteolin presents an H-bond interaction with LysB:58, where the ε-amino group (—CH_2_—NH_3_^+^) of LysB:58 donates the H to the 7-OH group of the luteolin A ring with 2.14 Å. At the same distance, an H-bond interaction with CysB:121 is present, where the NH_2_ group of the residue is the H donor to the 3′-OH group of luteolin. In the 3D model, the presence of a π-cation interaction between the luteolin C ring and the alkylammonium ion of LysB:225 is also observed (Figure 14). The luteolin–DENV E-3—SWCNT complex interaction also occurs in domain II of the DENV E-3 protein. The molecular interaction of this complex has close contact with TrpB:229, PheB:119, GlnB:120, GluB:123, LeuB:122, SerB:124, ThrB:223, AlaB:222, ThrB:224, ProB:227, LysB:225, and ThrB:226 residues (Figure 15).

### 3.3. Molecular Dynamics of DENV E-3–Flavone Complexes at Flavonoid Binding Sites

#### 3.3.1. Tropoflavin–DENV E-3 Complex

**Figure 16:**
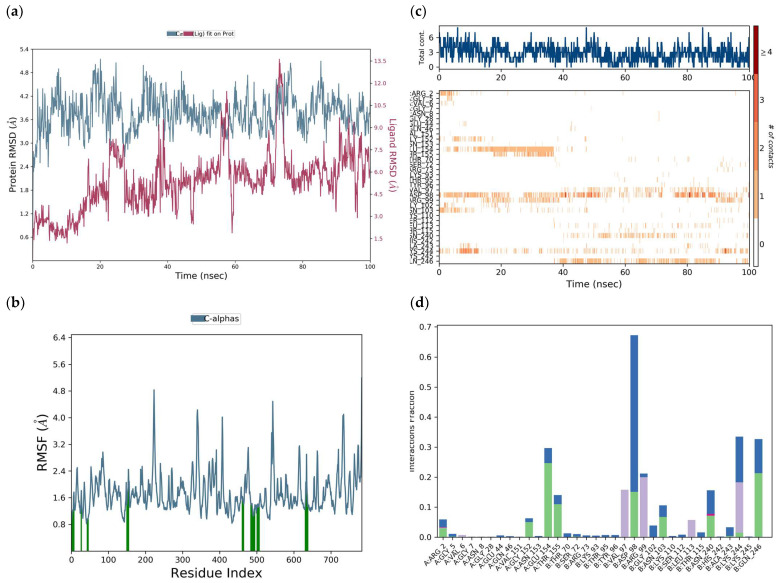
DENV E-3—Tropoflavin protein simulation trajectory with 100 ns. (**a**) RMSD protein. (**b**) RMSF protein. (**c**) DENV E-3—Tropoflavin interactions during the simulation trajectory. (**d**) Histogram of the DENV E-3—Tropoflavin interaction fraction 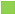 H-bonds, 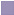 hydrophobic, 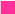 Ionic, 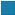 Water bridge.

The protein RMSD was between 2.8 and 3.8 Å through a simulation of 100 ns. The RMSD was 3.5 Å, but due to the interaction with the ligand, it increased to 5.2 Å at 23 ns and then decreased to 2.6 Å at 28 ns (Figure 16a). The RMSD of the ligand was between 1.5 and 7.5 Å, but due to its interaction with the protein, it presented deviations at 25 ns, 38 ns, 58, 75 ns, and 90 ns.

The RMSF of the protein (Figure 16b) was 1.45 Å but can be seen exhibiting fluctuations from 0.8 to 4.8 Å, along the whole receptor, representing the interaction of tropoflavin with the DENV E-3 protein. Protein residues interacting with the ligand are labeled with green vertical bars.

The total number of specific interactions that the DENV E-3 protein has with tropoflavin during the 100 ns simulation trajectory is presented in Figure 16c. Some residues such as Glu:154 show more than one interaction with tropoflavin, represented by a darker orange tone; however, the interaction was interrupted at 38 ns of the simulation trajectory. Meanwhile, the interaction with Asp:98 was maintained throughout the 100 ns with a slight interruption at 72 ns. The interaction with Lys:244 was maintained throughout the trajectory with repeated interruptions, and for Gln:246, the interaction was constant from 38 ns to 100 ns. In the 100 ns simulation trajectory, tropoflavin exhibits H-bond interactions with the residues ArgA:2, GlyA:152, GluA:154, ThrA:155, AspB:98, AsnB:103, AsnB:240, LysB:244, and GlnB:246. Hydrophobic interactions occurred with ValA:6, ValB:97, ArgB:99, LeuB:113, and LysB:244; water bridges occurred with ArgA:2, GlyA:5, GlyA:152, AsnA:153, GluA:154, ThrA:155, ThrB.70, AspB:98, ArgB.99, GlyB:102, AsnB:103, ThrB:115, AsnB:240, AlaB:243, LysB244, and GlnB:246; and ionic interactions occurred with ArgA:2 and AsnB:240 (Figure 16d). The interaction with AspB:98 was maintained for more than 68% of the simulation time. In the same way, it is important to highlight that tropoflavin presents one, two, or three types of interactions with several residues (Figure 16d, Table 1 and Table 3).

A main characteristic of the E protein is the presence of Asn153 in domain I and Asn67 in domain II [8]. In the molecular docking study between tropoflavin and the DENV E-3 protein, it was found that the interaction with Gly:152 at Δ*G* = −7.0 kca/mol presented close contact with the Asn153 residue, which is one of the conserved *N*-glycosylation sites involved in host cell binding and virus entry [8] and facilitates DENV infectivity [9]. Furthermore, glycosylation sites are related to virus production, pH sensitivity, and neuroinvasivity [59]. The molecular dynamics study of this interaction found that the interaction between tropoflavin and the Asn153 residue is mediated by a water bridge (Table 3).

#### 3.3.2. Baicalein–DENV E-3 Complex

**Figure 17:**
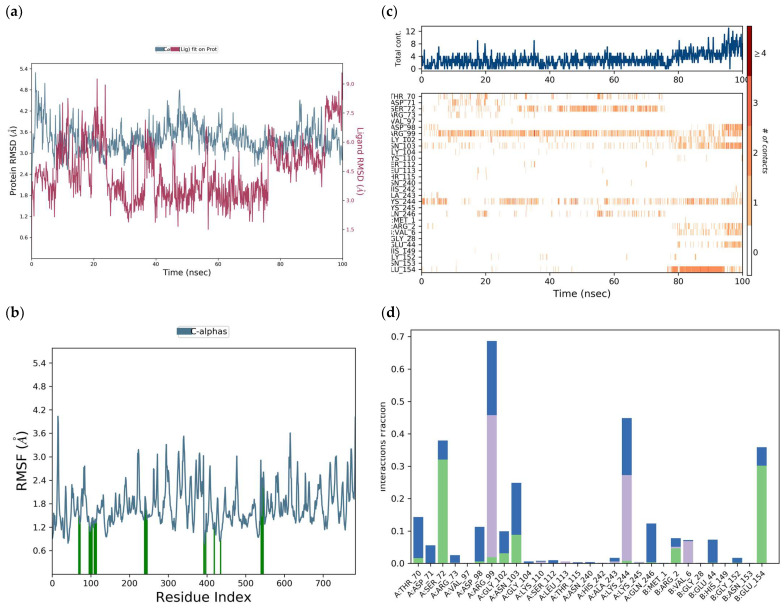
DENV E-3—Baicalein protein simulation trajectory with 100 ns. (**a**) RMSD protein. (**b**) RMSF protein. RMSD protein. (**b**) RMSF protein. (**c**) DENV E-3—Baicalein interactions during the simulation trajectory. (**d**) Histogram of DENV E-3—Baicalein interaction fraction 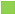 H-bonds, 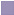 hydrophobic, 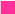 Ionic, 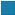 Water bridge.

The RMSD of the protein deviated between 3.0 and 3.3 Å during the 100 ns simulation trajectory of the DENV E-3–baicalein complex. The RMSD of the protein remained longer at approximately 3.3 Å; however, at 48 ns of the simulation trajectory, due to interaction with the ligand, it presented an increase to 4.9 Å (Figure 17a).

The RMSD of the ligand (right *Y* axis) indicates the stability of the ligand with respect to the protein and its binding site. The RMSD of the ligand remained for most of the simulation time at approximately 3.5 Å but, due to the interaction with the protein, increased to more than 9.0 Å at both 22 ns and 100 ns of the simulation trajectory. The peaks in the RMSF of the protein (Figure 17b) indicate the areas of the protein that fluctuate the most during the simulation. The RMSF of the protein was 1.46 Å.

From Figure 17c, at 100 ns, it is observed that baicalein in the interaction with Arg:99 was maintained for the longest time in the simulation trajectory. The interaction of baicalein with Lys:244 was maintained throughout the 100 ns simulation trajectory, presenting slight interruptions. Meanwhile, with Ser:72, the trajectory was interrupted at 78 ns and the interaction with Glu:154 remained constant from 78 to 100 ns. In the histogram of Figure 17d, it is observed that the interaction of baicalein with ArgA:99 was maintained for approximately 68% of the simulation time, and details of this hydrophobic π-cation-type interaction between ArgA:99 and the baicalein ring C are presented elsewhere (Supplementary Material Figure S3). It is important to highlight that ArgA:99 in addition to presenting hydrophobic interactions with baicalein also presents H-bond interactions and water bridges (Table 4).

Further, in Figure 17d, it is observed that baicalein exhibits hydrophobic interactions of the π-cation type with LysA:244, ValB:6, ArgB:2, LysA:110, and LeuA:113. π-cation interactions can occur between the B ring and, for example, residues LysA:244 and ArgB:2. Baicalein presents H-bond interactions with residues ThrA:70, SerA:72, AspA:98, ArgA:99, GlyA:102, AsnA:103, LysA:244, ArgB:2, and GluB:154. Likewise, its present water bridge interactions with residues ThrA:70, AspA:71, ArgA:73, AspA:98, ArgA:99, GlyA:102, AsnA:103, LysA:244, GlnA:246, ArgB:2, GlyB:152, and GluB:154. It is important to highlight that one, two, or three types of interactions are presented by the residues with baicalein in the flavonoid binding sites (Table 1 and Table 4).

In addition to the interaction of baicalein with protein E, in the binding sites of flavonoids, domain I of the B chain (residues 4–9, 151–154) and domain II of chain A (residues 98–103, 244–247) (Table 4), it also presents interactions with proteins NS3-NS2B and NS5, which could be responsible in part for its antiviral activity, according to other in silico studies [1,9]. Putri et al. (2023) [60] evaluated the cytotoxicity and anti-DENV 1–4 activity of baicalein and its synthetic derivatives in BHK-21 and Huh-7 cells and found that at high concentrations of 34 μM and 10.1 μM, baicalein did not exhibit cytotoxic effects. Furthermore, baicalein presented a significant reduction in DENV3 viral titers (approximately 1 log10-fold) in Huh-7 cells. Baicalein’s antiviral activity was found to be of the DENV serotype and cell-line-dependent. Hassandarvish et al. (2016) [9] at Δ*G* = −7.1 kca/mol found an interaction of baicalein with the Arg:2 residue of the DENV E-2 protein. Given the properties of some flavones, their antiviral activity (96–99%) is exerted by direct inactivation of free DENV-2 particles, as well as inhibition of both DENV-2 binding to the host cell and intracellular replication of the virus [1]. The Arg residue also plays an important role in the transfer of information between MTase and RdRp binding sites of the DENV-3 protein NS5 homodimer [61]. Furthermore, Arg forms H-bond interactions with allosteric inhibitors of DENV RdRp [62].

#### 3.3.3. Luteolin–DENV E-3 Complex

**Figure 18:**
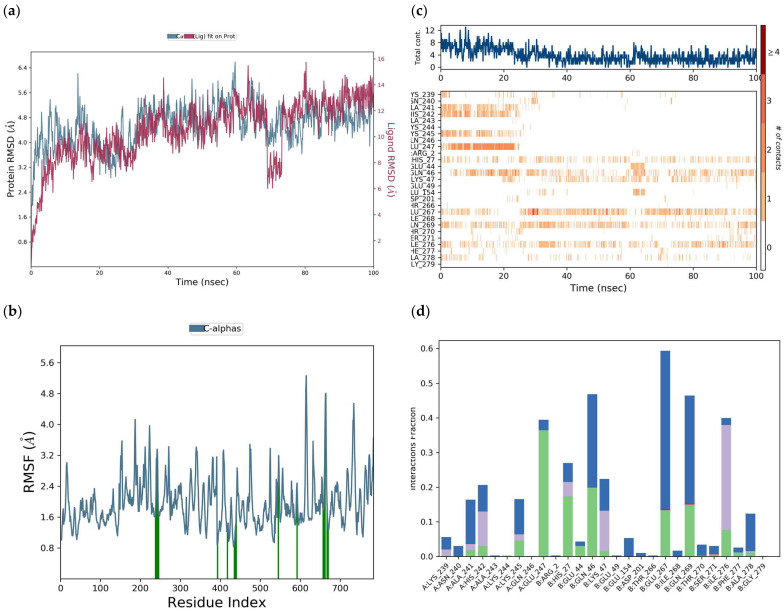
DENV E-3―Luteolin protein simulation trajectory with 100 ns. (**a**) RMSD protein. (**b**) RMSF protein. (**c**) DENV E-3―Luteolin interactions during the simulation trajectory. (**d**) Histogram of the DENV E-3―Luteolin interaction fraction 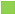 H-bonds, 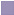 hydrophobic, 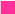 Ionic, 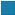 Water bridge.

The RMSD of the protein in a simulation trajectory of the DENV E-3–luteolin complex at 100 ns deviated between 2.0 and 4.1 Å. The RMSD was 4.1 Å but, due to the interaction with the ligand, increased to 6.7 Å at 58 ns and exhibited a slight decrease at 68 ns in the simulation trajectory (Figure 18a). The RMSF of the protein (Figure 18b) was 1.7 Å.

Throughout the 100 ns simulation trajectory, luteolin is observed to interact with Glu:267, followed by Gln:269, Gln:46, Ile:276, and His:27 with slight interruptions. The highest number of interactions was presented by luteolin with Glu:247 until 25 ns of the simulation trajectory, as well as with Lys:245, His:242, and Ala:241 (Figure 18c). Luteolin has H-bond interactions with AlaA:241, HisA:242, LysA:245, GluA:247, HisB:27, GluB:44, GlnB:46, LysB:47, GluB:267, GlnB:269, SerB:271, IleB:276, PheB:277, and AlaB:278. Ionic interactions occurred between luteolin and AlaA:241, GluB:267, GlnB:269, and SerB:271. In turn, hydrophobic interactions occurred with residues LysA:239, AlaA:241, HisA:242, LysA:245, HisB:27, LysB:47, and IleB:276 (Figure 18d). Water-bridge-type interactions occurred between luteolin and various residues such as LysA:239, AsnA:240, AlaA:241, HisA:242, LysA:245, GluA:247, HisB:27, GluB:47, GlnB:46, LysB:47, GluB:154, AspB:261, ThrB:266, GluB:267, IleB:268, GlnB:269, ThrB:270, SerB:271, IleB:276, PheB:277, and AlaB:278. Remarkably, the five residues GlnB:46, GluB:267, GlnB:269, IleB:276, and GluA:247 were maintained for more than 40% of the simulation time, and within these, the GluB:267 interaction was maintained for more than 60% of the trajectory, confirming the stability of the luteolin–DENV E-3 complex (Figure 18d).

Some H-bond-type interactions occurred between the 3′-OH group of the luteolin B ring and residues LysA:244 and LysA:245, where the 3′-OH group donates the H to the residues and in turn the 3′-OH group receives the H from residue HisA:242. The 4′-OH group also donates the H to the LysA:245 residue. The *4-oxo* group of the luteolin A ring can receive H from both residue GlnB:269 and the 5-OH group of ring B, and this group donates H to residue IleB:276. π-cation-type interactions occurred between the LysA:239 residue and the luteolin B ring.

Furthermore, water bridges can mediate various interactions, for example between the 5-OH group and the GluB:267 residue and between the 7-OH group and the AlaA:241 residue. It is also important to note that luteolin presents one, two, or three interactions with residues in some flavonoid binding sites (Table 1 and Table 5).

The presence and number of hydroxyl -OH groups play a fundamental role in the antiviral activity of flavonoids. Thus, the presence of hydroxyl groups at positions 5, 7, and 8 of ring A, the hydroxyl group at position 3 of ring C, and the hydroxyl groups at positions 3′and 4′ of ring B present a strong interaction of the flavonoid gossypetin with the envelope protein of the EBOV virus [63,64]. Luteolin, a tetrahydroxyflavone with the hydroxyl groups at the 3′,4′,5,7 positions, presented a binding affinity of −5.19 kcal/mol. Dwivedi et al. (2016) [65] performed virtual screening with various DENV NS3pro inhibitors and found that the binding affinity to a luteolin derivative was −7.00 kcal/mol. However, in studies by Ismail et al. (2017) [26], fisetin 3,3′,4’,7-tetrahydroxyflavone, a flavonol with the same hydroxyl number as luteolin, but varying only in one position of them, had a binding affinity of −7.4 kcal/mol.

More interactions with Glu, Lys, Gln, Ile, and Ala residues are present in the luteolin–DENV E-3 complexes. In the Flavone—DENV E-3 complexes, during the 100 ns simulation trajectory, it can also be observed that the RMSF of the different ligands evaluated, tropoflavin, baicalein, and luteolin, in the interaction with the DENV E-3 protein presents different ways of interacting with the protein.

**Figure 19:**
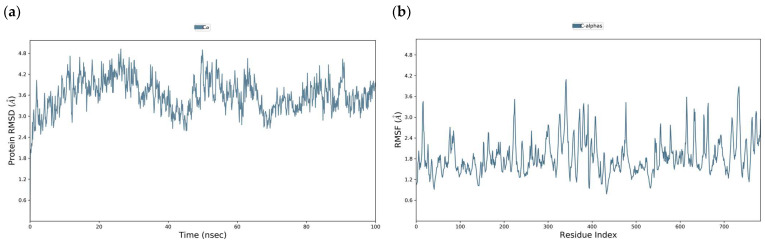
Apo DENV E-3 protein at 100 ns. (**a**) RMSD (**b**) RMSF.

**Figure 20:**
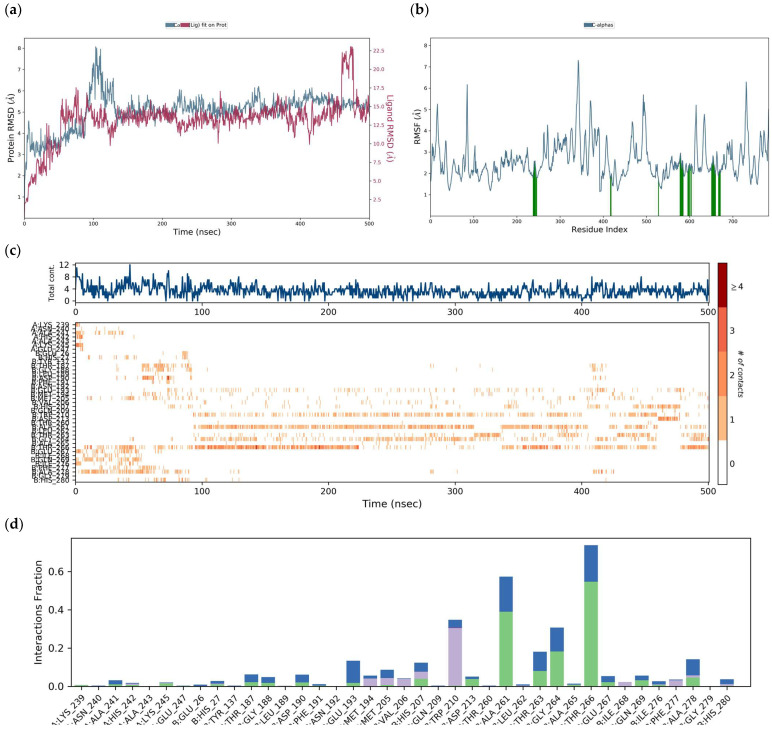
DENV E-3―Luteolin protein simulation trajectory with 500 ns. (**a**) RMSD protein. (**b**) RMSF protein. (**c**) DENV E-3―Luteolin interactions during the simulation trajectory. (**d**) Histogram of the DENV E-3―Luteolin interaction fraction 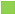 H-bonds, 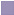 hydrophobic, 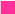 Ionic, 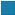 Water bridge.

The high RMSD values obtained in our study for the DENV E-3–tropoflavin, baicalein, and luteolin complexes suggest fluctuations of the protein itself. To verify the above, molecular dynamics simulations of the DENV E-3 protein without the ligand were performed. The high RMSD and RMSF values of the Apo protein suggest an inherent instability of the crystallographic protein in certain regions during the trajectory of 100 ns and 500 ns, as seen in Figure 19 and Figure 20, respectively. The RMSD of the Apo protein at 100 ns was 3.2 Å and the RMSF was 1.6 Å (Figure 19).

Racherla et al. (2022) [66] performed molecular dynamics studies of various DENV protein inhibitors on DENV -1, -2, -3 and -4 serotypes and reported high RMSD and RMSF values of the protein in the interaction with DENV-3. Similarly, Hossain et al. (2024) [67] performed molecular dynamics studies with baicalein and luteolin as anti-DENV-2 agents and found high RMSD and RMSF values in the interaction of these compounds with the NSP1 protein. For their part, Zong et al. (2023) [62] evaluated DENV NS5 RdRp inhibitors utilizing molecular dynamics studies. In a study of potential inhibitors of the DENV E-3 protein, it was found that in an MD trajectory of 100 ns, chlorogenic acid interacted with 27 amino acids [65].

Based on the MD results of the interaction of the DENV E-3–luteolin complex (Figure 18) at 100 ns, the simulation of the same interaction was performed for 500 ns. The RMSD of the DENV E-3 protein in the DENV E-3–luteolin complex at 500 ns was 4.8 Å and the RMSF was 2.6 Å. The increase in both RMSD and RMSF values with respect to the RMSD and RMSF of the DENV E-3–luteolin complex at 100 ns was observed to be 4.1 Å and 1.7 Å, respectively (Figure 20).

H-bond interactions with residues AlaA:241, HisA:242, LysA:24, and HisB.27 and water bridges with residues AlaA:241, ThrB:266, GluB:267, GlnB:269, IleB:276, and AlaB:278 at both 100 ns and 500 ns of the simulation trajectory are observed in molecular dynamics studies of the luteolin–protein DENV E-3 complex.

Among the mechanisms through which flavonoids are thought to exert their antiviral activity are obstructing virus attachment and entry into the host cell, altering the various stages of viral RNA replication, and preventing them from invading other healthy cells [64,68,69]. The noncompetitive inhibition of host furin proprotein convertase activity is the main mechanism through which luteolin exerts its anti-DENV action. The reduction or inhibition of virus replication is caused by the cleavage of the prM protein since it interrupts the maturation of the virus, resulting in immature viral particles [70]. Zandi et al. (2011) [71] found that fisetin inhibited the replication of DEV-2 (IC_50_: 55 μg/mL). In that study, they concluded that fisetin possibly does not affect virus binding to the cell, but that its anti-DENV activity is due to fisetin interfering directly with virus RNA replication. Flavonoids may have anti-dengue activity by interacting with several sites of the dengue virus [24,25].

## 4. Limitations and Perspectives

Although in silico studies on the anti-DENV action of flavones are few, the results of this work support the development of valid strategies in the search for natural agents with anti-DENV E-3 action. Based on experimental studies on the anti-DENV activity of the flavones evaluated, it is recommended to conduct in vitro studies that validate the results found in this study considering the molecular interactions of Flavone—DENV E-3, flavone–SWCNT, and Flavone—DENV E-3-SWCNT as pathways for finding new drugs with anti-DENV action.

## 5. Conclusions

Molecular interactions between the flavones evaluated and the DENV E-3 protein are mainly through H-bonds, hydrophobic bonds, and water bridges. Molecular docking studies between the flavones tropoflavin, baicalein, and luteolin and the DENV E-3 protein as anti-DENV agents allowed me to verify that in the tropoflavin–DENV E-3 complex, there is an H-bond interaction with GlyA:152 which has close contact with the Asn153 residue, which is one of the conserved *N*-glycosylation sites and which is involved in the binding and entry of the virus into the cell. In the same way, tropoflavin, baicalein, and luteolin present H-bond interactions with residues found in domain II of chain A where the fusion peptide region is located. The interaction of the Flavone—DENV E-3—SWCNT complex with the flavones with anti-dengue activity occurred at an energy value of −11.77 kcal/mol. This interaction occurred with domain II of the protein. Molecular dynamics studies of the flavone—DENV E-3 interaction in each of the complexes evaluated allowed me to infer that the number of hydroxyls present in the flavone molecule, as well as differences in protein folding, may be factors to consider in the stability of the Flavone—DENV E-3 protein interaction in light of the RMSD and RMSF values obtained in this study. In Flavone—DENV E-3 interactions, in addition to hydroxyl groups, amine, amide, and carboxylic acid groups are also involved. The results obtained here provide valid information on anti-DENV E-3 activity and can support future in vitro studies.

## Figures and Tables

**Table 1:** Flavone—DENV E-3 H-bond interactions at flavonoid binding sites.

Flavone	Δ*G* (Kcal/mol)	H-Bond Interaction with DENV E-3	Residue/Flavone Interaction Functional Groups	Distance (Å)
*Tropoflavin*	−7.0	GlyA:152	C=O―8-OH	2.37
	−4.49	LysA:245	NH_3_^+^―7-OH	2.42
*Baicalein*	−3.3	ArgA:99	NH_2_―C=O	2.45
*Luteolin*	−5.19	LysA:245	C=O―3’-OH	2.45

**Table 2:** Interactions of π-cation–Flavone—DENV E-3 at flavonoid binding sites.

Flavone	Δ*G* (kcal/mol)	π-Cation Interaction	Residue/Flavone Interaction Functional Groups	Distance (Å)
*Baicalein*	−6.4	LysA:244	NH_3_^+^—A ring	3.72

**Table 3:** Interaction types of residues in close contact with tropoflavin.

Interaction Type	Residue
H-bond	AsnB:103, GlyA:152, GluA:154, ArgA:2, AspB:98, AlaB:243, LysB:244
Water bridge	AsnB:103, GlyB:102, GlyA:152, AsnA:153, GluA:154, ArgA:2, GlyA:5, AspB:98, AlaB:243, LysB:244
Ionic	ArgA:2
π-cation	ValA:6, LysB:244, ValB:97

**Table 4:** Interaction types of residues in close contact with baicalein at flavonoid binding sites.

Interaction Type	Residue
H-bond	ArgA:99, GluB:154, LysA:244, AspA:98, GlyA:102, AsnA:103
Water bridge	ArgA:99, GluB:154, GlnA:246, GlyB:152, AspA:98, GlyA:102, AsnA:103
π-cation	ArgA:99, LysA:244, ValB:6

**Table 5:** Interaction types of residues in close contact with luteolin at some flavonoid binding sites.

Interaction Type	Residue
H-bond	LysA:245, HisA:242, AlaA:241, GlnA:246, AlaB:278, GluB:267, GlnB:269, IleB:276, GluA:247
Water bridge	LysA:245, HisA:242, AsnA:240, AlaA:241, GlnA:246, AlaB:278, GluB:267, IleB:268, LysA:239, GlnB:269, IleB:276, GluA:247
Ionic	AlaA:241, GluB:267, GlnB:269
π-cation	LysA:245, HisA:242, AlaA:241, AlaB:278, LysA:239, IleB:276

## Data Availability

Data are contained within the article and Supplementary Materials.

## Supplementary Materials

The supporting information can be downloaded at https://www.mdpi.com/article/10.3390/v17040525/s1. Figure S1. A. 7,8-dihydroxyflavone. B. 5,6,7-trihydroxyflavone. C. 5,7,3´,4´-tetrahydroxyflavone. Figure S2. Binding energy histogram of DENV E-3—SWCNT interaction. Figure S3. π-cation interaction between ArgB:9 and baicalein at 32% of the simulation time. Table S1. Free energy of binding of luteolin–DENV E-3 interaction with Autodock 4.0.
