# Supplementary material for: Modeling and Molecular Dynamics Studies of Flavone―DENV E-3 Protein―SWCNT Interaction at the Flavonoid Binding Sites
Source: Viruses. 2025 Apr 4;17(4):525. doi: 10.3390/v17040525 (PMC12031533; doi:10.3390/v17040525)
Supplement: Supplementary file 1 [file viruses-17-00525-s001.zip › viruses-3445429-supplementary.pdf]

Supplementary Information

Modeling and Molecular Dynamics Studies of Flavone—DENV E-3 Protein—SWCNT Interaction at the Flavonoid Binding Sites

Cecilia Espíndola

University of Seville, C/Profesor García González 1, 41012 Seville, Spain.

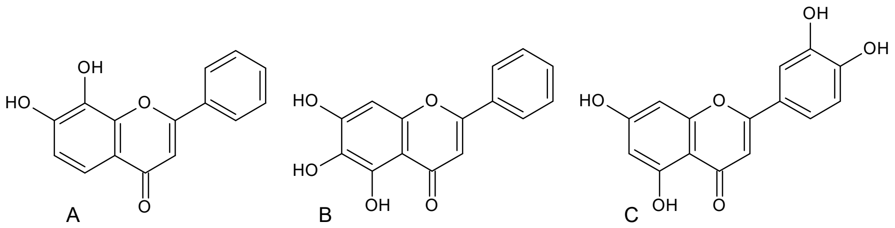

Supplementary Material. Figure S1. A. 7,8-dihydroxyflavone. B. 5,6,7-trihydroxyflavone. C. 5,7,3′,4′-tetrahydroxyflavone.

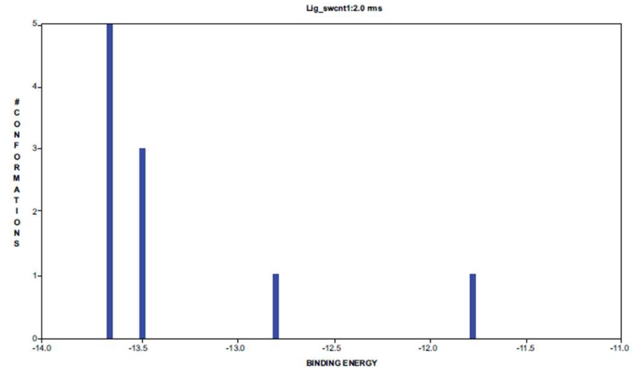

Supplementary Material. Figure S2. Binding energies histogram of DENV E-3— SWCNT interaction.

Energy histogram from blind coupling performed with Autodock showing four clusters. From left to right, the first cluster groups the binding energies -13.5, -13.65, -13.61 and -13.59 kcal/mol; in this cluster are residues corresponding to domain II of the DENV E-3 protein. The next cluster groups the energies -13.47, -13.44 and -13.48 kcal/mol, where residues corresponding to domain I are found. The third cluster corresponds to the energy value -12.79 kcal/mol and is located between domain II and III. The last cluster corresponds to the energy value -11.77 kcal/mol.

Supplementary Material. Table S1. Free Energy of Binding of Luteolin—DENV E-3 interaction with Autodock 4.0

Clustering	Binding E	Ligand effi	Ki mM	Intermol E	Desolv E	Electrostatic E	Total internal	Torsional E	Unbound E	clRMSD	RefRMSD
1	-4.61	-0.22	0.42	-6.1	-5.78	-0.32	-1.95	1.49	-1.95	0	53.563
2	-3.07	-0.15	5.66	-4.56	-4.46	-0.1	-1.97	1.49	-1.97	0	43.888
3	-3.95	-0.19	1.26	-5.45	-5.42	-0.03	-1.96	1.49	-1.96	0	53.204
4	-2.98	-0.14	6.54	-4.47	-4.36	-0.11	-1.97	1.49	-1.97	0.61	44.32
5	-3.87	-0.18	1.45	-5.36	-5.15	-0.21	-1.73	1.49	-1.73	0	52.184
6	-4.9	-0.23	0.255	-6.39	-6.39	-0.01	-1.97	1.49	-1.97	1.75	76.32
7	-3.24	-0.15	4.22	-4.73	-4.72	-0.01	-1.95	1.49	-1.95	0	45.435
8	-5.5	-0.26	0.0925	-6.99	-6.79	-0.2	-1.89	1.49	-1.89	0	77.106
9	-4.06	-0.19	1.06	-5.55	-5.4	-0.15	-1.84	1.49	-1.84	0	52.823
10	-5.19	-0.25	0.157	-6.68	-6.39	0.29	-1.93	1.49	-1.93	0	25.933

Free Energy of Binding [= (1) +(2) + (3) – (4)]

(1) Final intermolecular energy
(2) Final total internal energy vdW + H-bond + desolv E + Electrost. E
(3) Torsional Free Energy
(4) Unbound System Energy

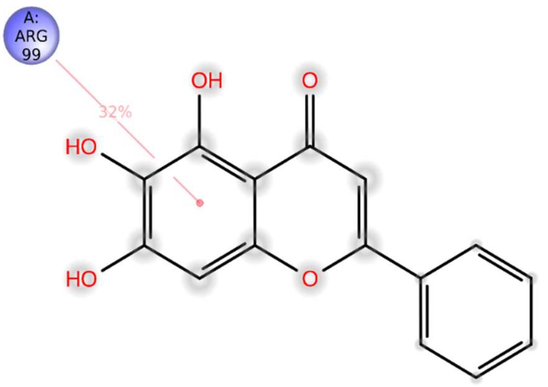

Supplementary Material. Figure S3. π-cation interaction between ArgB:9 and Baicalein with 32% simulation time.
